# Age and Positional Effect on the Anterior Chamber Angle: Assessment by Ultrasound Biomicroscopy

**DOI:** 10.1155/2013/706201

**Published:** 2013-04-23

**Authors:** Nicholas P. Bell, Kundandeep S. Nagi, Ricardo J. Cumba, Alice Z. Chuang, David A. Lee, Thomas C. Prager, Kavita Rao, Robert M. Feldman

**Affiliations:** ^1^Ruiz Department of Ophthalmology and Visual Science, The University of Texas Medical School at Houston, 6431 Fannin Street, MSB 7.024, Houston, TX 77030, USA; ^2^Robert Cizik Eye Clinic, 6400 Fannin Street, Suite 1800, Houston, TX 77030, USA; ^3^Department of Ophthalmology, The University of Texas Health Science Center at San Antonio, 7703 Floyd Curl Drive, Mail Code 6230, San Antonio, TX 78229, USA; ^4^Ophthalmology Department, University of Puerto Rico, Medical Sciences Campus, P.O. Box 365067, San Juan, PR 00936, USA; ^5^Houston Eye Associates, 2855 Gramercy Street, Houston, TX 77025, USA

## Abstract

*Purpose*. To investigate age- and position-related changes of anterior chamber angle anatomy in normal, healthy eyes. *Patients and Methods*. Thirty subjects were separated into a younger and older cohort. The superior and inferior anterior chamber angles of the eyes were measured in supine and sitting positions by ultrasound biomicroscopy (UBM) with bag/balloon technology. Statistical analysis was used to evaluate positional and age-related changes in angle morphology. *Results*. In the younger cohort, no location or positional differences in angle anatomy were observed. In the older cohort, the inferior quadrant was significantly narrower than the superior quadrant (*P* = 0.0186) in the supine position. This cohort also demonstrated an interaction effect between position and location. In the older cohort, the angle was deeper inferiorly while the subject was sitting but was deeper superiorly while the subject was supine. *Conclusion*. Comparison of positional variations in anterior chamber angle anatomy as measured by UBM has recently become possible. This study found that age-related positional changes in the anterior chamber angle anatomy exist in normal healthy eyes.

## 1. Introduction

Ultrasound biomicroscopy (UBM) provides noninvasive high-resolution *in vivo *imaging of the anterior segment. While anterior tissues are readily visualized by conventional methods (e.g., slit-lamp biomicroscopy and gonioscopy), structures posterior to the iris are hidden due to absorption of light by the iris pigment epithelium. Such structures may also be undetected by anterior segment optical coherence tomography (ASOCT), given the inability of light to penetrate the iris pigment epithelium. B-scan ultrasonography, though extremely useful in imaging posterior ocular structures, is also not ideal for ciliary body imaging due to the near-field artifact and poorer resolution obtained with lower frequencies. However, the higher frequency ultrasound waves, utilized by UBM, are not obstructed by pigmented tissue and give the required resolution. Thus, UBM can be used to image not only the anterior chamber angle but also the ciliary body, peripheral lens, zonules, and the posterior chamber of the eye [1]. UBM has been demonstrated to be useful in evaluating the anterior segment of eyes with the primary angle closure spectrum of disorders [2]. It also may be used to elucidate the mechanism of malignant glaucoma, pigmentary glaucoma, and anterior scleral disease [3–5]. Therefore, UBM has become the current standard for imaging abnormalities of ciliary body position, as are found in plateau iris configuration and annular choroidal effusions. 

UBM technology is now readily available and simple to use with the development of a water-filled bag (ClearScan Cover, ESI, Inc., Plymouth, MN), replacing traditional open-shell immersion techniques. The single-use, sterile bag snugly fits over the distal end of the probe to form a watertight seal, which when pushed against the eye creates balloon-like positive pressure [6]. The tip of the UBM probe does not contact ocular structures, thus overcoming near-field artifact as well as increasing patient comfort. A distinct advantage of this technique is the ability to perform UBM examinations with the subject in any position [6]. 

Historically, as used clinically, UBM examinations have only been possible in the supine position due to the requirement of an open-shell immersion technique to overcome the acoustic near-field artifact inherent to ultrasonic imaging. There have been scattered reports in the literature of positional measurements using UBM [7–10]. However, the effects of age and supine to sitting positional change on anterior chamber anatomy in normal subjects are not known. Changes in laxity of the irido-lenticular-zonular (ILZ) apparatus with age theoretically may lead to alterations of the anterior chamber angle configuration that may be identified and measured with UBM. This study is designed to investigate age- and position-related changes of anterior chamber angle anatomy in normal, healthy eyes.

## 2. Patients and Methods

This research was approved by the Committee for the Protection for Human Subjects at The University of Texas Health Science Center at Houston. The protocol and procedures adhered to the tenets of the Declaration of Helsinki, and the study was HIPAA compliant.

### 2.1. Study Population

Subjects were initially considered for participation if they were between 18 and 30 years of age (younger cohort) or older than 45 years of age (older cohort) and could tolerate both the supine and sitting positions for at least 15 minutes. Informed consent was obtained, and a screening history and examination were performed consisting of slit-lamp biomicroscopy, applanation tonometry, and gonioscopy. Subjects were considered eligible if the following criteria were met: open angles (Schaffer grade 3-4), intraocular pressure (IOP) 8–21 mm Hg, and no ocular history other than refractive error. If both eyes were eligible, the right eye of each participant was selected as the study eye.

### 2.2. UBM Imaging

The superior and inferior anterior chamber angles were imaged at the limbus using the VuMax II UBM (Sonomed, Lake Success, NY) in high resolution mode with a 35 MHz probe tipped with a ClearScan Cover under topical anesthesia as previously described [6]. Of note, the technique was modified by filling the bag to the lower aspect of the sealing collar instead of to the top end. Thus, the bag had a lower internal positive pressure than the eye to minimize compression of the ocular structures. With the probe's orientation line facing the cornea, the UBM probe was held perpendicular to the segment of angle being measured.

Images of the anterior chamber angle at superior and inferior quadrants were taken in sitting and supine positions in a dark room in a randomized fashion. Three sets of images were taken of each participant under each testing condition.

### 2.3. Measurement

**Figure 1:**
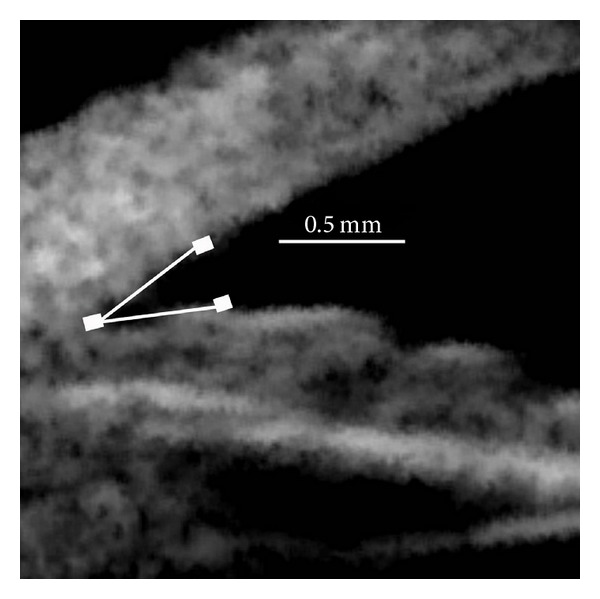
The anterior chamber angle (ACA) is the angular measurement (in degrees) created by two 500 micron line segments originating at the iris root, extending along the posterior cornea and anterior iris surface.

The primary outcome measure was the anterior chamber angle (ACA, in degrees) formed from the angle created by two 500 micron line segments originating at the iris root, extending along the posterior cornea and anterior iris surface (see Figure 1). Three images were obtained for each quadrant (superior and inferior) in each position (sitting and supine), with the largest angle measurement (for consistency) recorded in each test condition. To validate the use of the largest angle measurement, a post hoc analysis using the mean of the 3 measurements was performed.

### 2.4. Sample Size Calculation and Statistical Analysis Methods

A sample size of 14 subjects per group was determined to be sufficient to detect a mean difference of 5 degrees between age groups for each condition at a 5% significance level and 80% power assuming that the angle was normally distributed with standard deviation of 4.5 degrees. Hence, 15 subjects were recruited per group to allow for potential loss during testing. 

Descriptive statistics, mean and standard deviation, were calculated for each condition by age group. The Kolmogorov-Smirnov test was performed to validate that the ACAs were normally distributed. A mixed-effect model was used to compare the overall ACA between age groups and the interaction effect of location and position within each cohort. The paired *t*-test was used to compare the effect of condition changes (position: seated versus supine; location: superior versus inferior) on ACA within participants for each age group. The mean difference in ACA between younger and older age groups for each condition (i.e., position and angle location) was computed, and a two-sample *t*-test was used to determine significant changes. The analyses were performed using SAS v9.2 (SAS Institute Inc., Cary, NC). *P* values < 0.05 were considered statistically significant.

## 3. Results

A total of 30 participants were enrolled in the study, with 15 in each age cohort. All enrolled participants completed the study. The mean age in the older cohort was 57.4 ± 6.7 (range: 45–70) years and in the younger cohort was 25.7 ± 0.7 (range: 24–27) years. There were 10 (67%) males in the older cohort and 9 (60%) males in the younger cohort. Twelve participants (80%) were white, two Asian, and one black in the older cohort while 10 participants (66%) were white, four Asian, and one black in the younger cohort. The Kolmogorov-Smirnov test showed that ACAs were normally distributed (*P* = 0.1500). Overall, the mean ACA was 37.4 ± 4.8 degrees for the older cohort and 41.0 ± 5.0 degrees for younger cohort. This difference of 3.6 degrees was statistically significant (*N* = 15 × 4 = 60 angles; *P* = 0.0136 using a mixed-effect model). In the sitting/superior and inferior/supine conditions, there was a statistically significant difference between age groups, with the younger group having larger angles. 

### 3.1. Location (Superior versus Inferior Angle)

Except for the supine position in the older cohort, where the inferior quadrant was significantly narrower than the superior quadrant (*P* = 0.0186), there were no statistically significant differences (Table 1) in ACA.

### 3.2. Position (Sitting versus Supine)

There were no statistically significant changes in the ACA regardless of the angle location or age cohort. However, there was a trend for deepening of the superior angle (*P* = 0.1322) and narrowing of the inferior angle (*P* = 0.0583) upon changing from the supine to the sitting position in the older cohort. 

### 3.3. Position (Sitting versus Supine) and Location (Superior versus Inferior) Combined Effect

In the younger cohort there was no interaction between location and position; however, an interaction effect was observed in the older group. This observation was verified by a mixed-effect model with location, position, and their interaction as the fixed effects for each cohort. The interaction between position and location was not significant for the younger cohort (*P* = 0.9002) but was significant in older cohort (*P* = 0.0130). In older cohort, the angle was deeper *inferiorly* while the participant was *sitting* but was deeper *superiorly* while the subject was *supine*.

## 4. Discussion

The development of the water-filled probe cover has increased the comfort and versatility of UBM examination of the ACA. While previous studies have examined the structural changes in the ACA [7–11], this is the first study to address whether positional changes in angle measurements vary with age. 

In this study, the ACA of the older cohort was overall 4.5 degrees narrower than that of the younger cohort (mean ACA of 37.4 ± 4.8 degrees in the older cohort versus 41.0 ± 5.0 degrees in the younger cohort; *P* = 0.0136). This supports the well-documented literature regarding narrowing of the anterior chamber angle with age [11–14], the cause of which may be from continuous growth and increased thickness of the crystalline lens throughout life [15]. 

There is debate regarding which quadrant is the narrowest. Esaki et al. reported with UBM, in agreement with clinically accepted gonioscopic findings, that the superior angle is the narrowest while the inferior is the deepest [12]. However, Friedman et al. suggest inconsistency between sitting gonioscopic and supine UBM findings, reporting that the inferior angle is the narrowest when measured by UBM in the supine position [11]. In the current study, for the younger cohort, there was no difference between the superior and inferior angle width regardless of position. In the older cohort, the inferior angle was narrower than the superior angle in the supine position (inferior angle narrower by 2.1 ± 3.0 degrees; *P* = 0.0186), in contrast to the sitting position, where no significant difference was found between the superior and inferior angle widths. 

**Figure 2:**
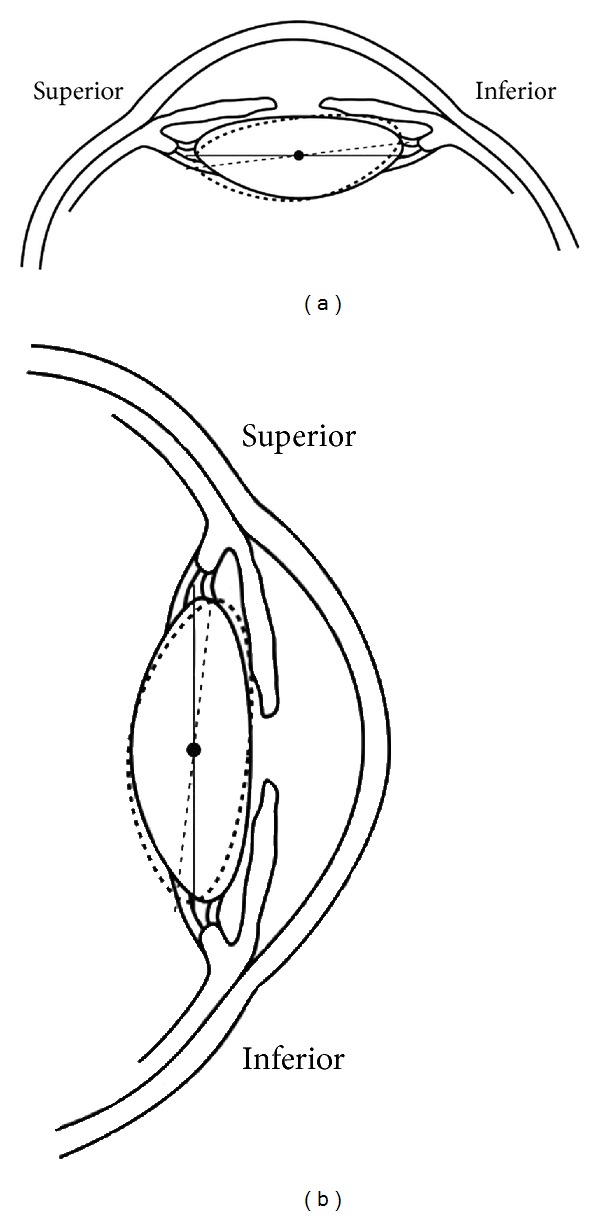
Tilting of the lens in the supine (a) and sitting (b) positions during ultrasound biomicroscopy imaging in the older and younger cohorts. Dashed line = older cohort. Solid line = younger cohort.

Position has been hypothesized to affect ACA width. Ishikawa et al. suggested that supine positioning would allow the lens to fall backwards, deepening the angle [16]. In addition, Friedman et al. reported that supine UBM and sitting gonioscopy are poorly correlated and theorized that increased gravitational forces on the lens in the sitting position result in a deeper inferior ACA [11]. Until the current study, conclusions comparing UBM to gonioscopy were difficult because they were not generally performed in the same position. In this study, no significant difference in the marginal ACA with positional change was noted in either cohort. However, there was an interaction observed in the *older cohort*: the magnitude of deepening in the superior angle when moving from the sitting to supine position was matched by the magnitude of narrowing of the inferior angle with the same positional change. This phenomenon may be consistent with Friedman's theory [11] and may suggest positional tilting of the lens (Figure 2).

In the current study, there was a positional change in angle measurements in the older cohort, which was not found in the younger cohort. One potential explanation for this age-related difference is that the irido-lenticular-zonular (ILZ) apparatus may develop laxity with age with resultant tilting of lens position as described earlier and detailed in Figure 2. 

This study has several limitations. Due to limitations of 35 mHz UBM image resolution, accurate identification of the scleral spur was not possible. Commonly used angle measurements, angle opening depth (AOD) and angle recess area (ARA), require a reproducible scleral spur; therefore, the iris root was used as the apex of the angle. This should not affect our results since this study did not include eyes with closed angles. Additionally, previous reports evaluating anterior chamber anatomy with UBM have suggested that the placement of a cup to hold liquid over the eye may indent the sclera and alter angle anatomy [17]. Using the bag/balloon technology, there is no hard shell or cup to artifactually indent the sclera/cornea. It is important to note that the internal bag pressure must be lower than the IOP of the patient's eye to avoid denting the cornea. Bag pressure can be changed by adjusting the amount of water in the bag and the probe insertion depth. One other potential weakness is the use of the largest of 3 repeated angle measurements as data. To evaluate this, a post hoc analysis using means of the 3 angle measurements was performed and resulted in identical conclusions. 

Angle anatomy changes with position in older participants, while it remains consistent in younger participants. This age-related difference may help explain inconsistencies in the literature on relative angle widths within an eye. Understanding normal angle anatomic changes may assist us in understanding the pathophysiologic events in diseases related to anterior chamber angle anatomy. 

## Figures and Tables

**Table 1:** Comparing positions at each location in each cohort.

	Sitting	Supine	Sit − Supine^§^
Younger^†^			

Superior	41.3 ± 5.7*	41.5 ± 4.4	−0.2 ± 5.7
Inferior	40.6 ± 6.6	40.5 ± 3.3*	0.1 ± 7.1
Superior − Inferior^‡^	0.7 ± 7.0	1.0 ± 4.7	

Older			

Superior	36.8 ± 4.5	38.3 ± 5.2	−1.5 ± 3.6
Inferior	38.1 ± 4.4	36.2 ± 5.4	1.9 ± 3.6
Superior − Inferior^‡^	−1.3 ± 3.4	Narrower by 2.1 ± 3.0**	

^†^Comparing mean ACA between age cohorts using two sample *t*-test.

^‡^Comparing mean ACA between superior and inferior quadrants using paired *t*-test.

^§^Comparing mean ACA between seated and supine positions using paired *t*-test.

*Statistically significantly different between age cohorts.

**Statistically significantly different between superior and inferior quadrants within older cohort.
